# Acute Phosphate Nephropathy Following Oral Sodium Phosphate Bowel Preparation

**DOI:** 10.7759/cureus.112061

**Published:** 2026-07-04

**Authors:** Enes Oztop

**Affiliations:** 1 Internal Medicine, Ankara University, Ankara, TUR

**Keywords:** acute kidney injury, acute phosphate nephropathy, bowel preparation, purgative-induced kidney injury, sodium phosphate, temporary hemodialysis

## Abstract

Acute phosphate nephropathy (APN) is a rare but important cause of acute kidney injury (AKI), most commonly associated with the use of oral sodium phosphate bowel preparations administered prior to colonoscopy. In this report, we describe the case of a 60-year-old female patient who developed severe AKI necessitating temporary hemodialysis after bowel preparation with a phosphate-containing laxative. Quick identification, cessation of nephrotoxic agents, aggressive intravenous hydration, and supportive hemodialysis therapy facilitated gradual renal recovery. This case points out the importance of heightened awareness of APN, particularly in patients with multiple predisposing factors.

## Introduction

Acute phosphate nephropathy (APN) is a rare but potentially serious cause of acute kidney injury (AKI) that occurs following exposure to oral sodium phosphate (OSP) bowel preparations, most commonly administered before colonoscopy. The disorder is characterized by excessive intestinal phosphate absorption, resulting in transient hyperphosphatemia, hypocalcemia, and intratubular precipitation of calcium phosphate crystals. Crystal deposition within the renal tubules can induce tubular obstruction, direct epithelial injury, and an inflammatory response, which may lead to acute or permanent impairment of kidney function [1,2].

Risk factors include chronic kidney disease, advanced age, female sex, dehydration, diabetes mellitus, hypertension, and concurrent use of nephrotoxic medications such as non-steroidal anti-inflammatory drugs (NSAIDs) and renin-angiotensin-aldosterone system (RAAS) inhibitors [1,3,4].

Although awareness of the nephrotoxic potential of OSP preparations has increased, they remain in clinical use, and APN continues to be underrecognized. Early recognition is important because prompt withdrawal of the offending agent and supportive management may improve renal recovery and help prevent irreversible kidney damage [1,3].

## Case presentation

A 60-year-old woman presented to the emergency department with a two-day history of anuria, fatigue, nausea, and flank pain. Her medical history included type 2 diabetes mellitus, hypertension, hyperlipidemia, and migraine. Her regular medications included metformin, ramipril, atorvastatin, and intermittent use of NSAIDs, specifically naproxen and dexketoprofen trometamol, with the last NSAID intake occurring approximately 10 days before admission. Her baseline renal function was normal, with a serum creatinine level of 0.69 mg/dL measured two months before admission.

Two days before the presentation, she underwent bowel preparation for a planned colonoscopy using a phosphate-containing laxative and reported reduced oral intake during this period. On examination, she was alert and oriented. Her blood pressure was 144/80 mmHg, pulse rate was 77 bpm, and oxygen saturation was 97% on room air, with no evidence of volume overload or pulmonary congestion. Cardiovascular, respiratory, and abdominal examinations were unremarkable.

Initial laboratory investigations revealed severe AKI, and Table 1 summarizes the results:

**Table 1:** Laboratory findings at hospital admission.

Parameter	Conventional unit	SI unit	Reference range (conventional/SI)
Serum creatinine	5.34 mg/dL	472 µmol/L	0.6-1.2 mg/dL/53-106 µmol/L
Blood urea nitrogen (BUN)	48 mg/dL	17.1 mmol/L	7-20 mg/dL/2.5-7.1 mmol/L
Serum sodium	122 mEq/L	122 mmol/L	135-145 mEq/L/135-145 mmol/L
Serum phosphate	6.4 mg/dL	2.07 mmol/L	2.5-4.5 mg/dL/0.81-1.45 mmol/L
Serum uric acid	8.8 mg/dL	523 µmol/L	3.5-7.2 mg/dL/208-428 µmol/L
Serum magnesium	1.67 mg/dL	0.69 mmol/L	1.7-2.2 mg/dL/0.70-0.91 mmol/L
Serum potassium	4.4 mEq/L	4.4 mmol/L	3.5-5.1 mEq/L/3.5-5.1 mmol/L

**Figure 1:**
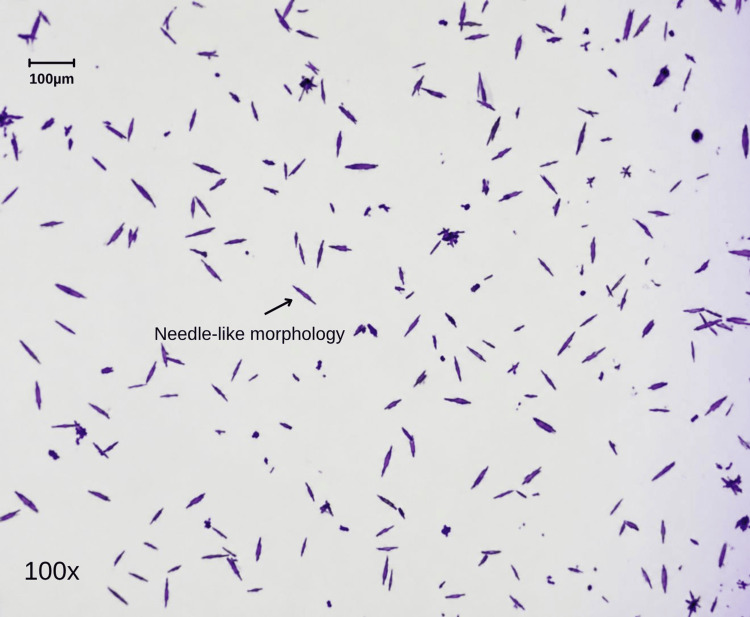
Urine sediment microscopy under non-polarized light. Urine sediment microscopy under non-polarized light (100× magnification). Numerous elongated needle-shaped crystals suggestive of phosphate crystalluria are observed. The arrow indicates a representative elongated needle-shaped phosphate crystal. Scale bar = 100 µm.

**Figure 2:**
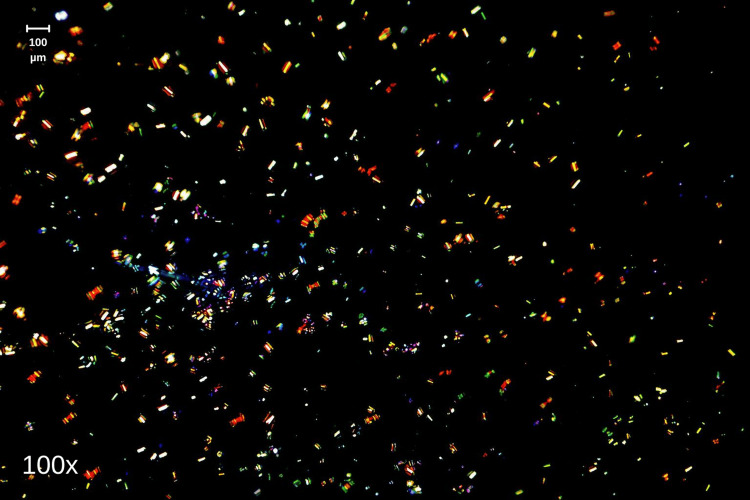
Polarized light microscopy of urine sediment. Polarized light microscopy of urine sediment (100× magnification). Numerous elongated crystals demonstrating birefringence with a multicolored appearance under polarized light are observed. Scale bar = 100 µm.

Microscopic examination of the urine sediment revealed abundant elongated phosphate crystals on non-polarized microscopy, with distinct birefringence observed under polarized light microscopy (Figures 1-2).

A non-contrast computed tomography (CT) scan of the abdomen and pelvis revealed normal-sized kidneys with preserved contours and no evidence of hydronephrosis or urinary tract obstruction. Renal ultrasonography confirmed a normal collecting system without hydronephrosis.

The differential diagnosis included acute tubular necrosis secondary to volume depletion, pre-renal azotemia, drug-related nephrotoxicity (ACE inhibitor and NSAID exposure), acute interstitial nephritis, obstructive uropathy, and APN.

Given the recent history of phosphate-based bowel preparation, hyperphosphatemia, urinary phosphate crystals, and the absence of urinary tract obstruction, APN was considered the most likely contributing cause of AKI. Renal biopsy was not performed because the patient demonstrated rapid clinical and biochemical improvement with supportive management, making an invasive diagnostic procedure unnecessary. Accordingly, APN was considered a presumptive rather than histologically confirmed diagnosis. The temporal association between phosphate exposure, hyperphosphatemia, phosphate crystalluria, and subsequent renal recovery further strengthened the clinical suspicion of APN.

The treatment involved the cessation of all potential nephrotoxic agents, including NSAIDs, metformin, and diuretics. The patient received aggressive intravenous hydration with isotonic saline, phosphate-binding therapy, correction of electrolyte imbalance, and close monitoring of urine output and renal function. No significant metabolic acidosis or hyperkalemia was observed. However, despite adequate volume replacement and supportive treatment, the patient remained persistently anuric for approximately five consecutive days, including the two days preceding hospital admission and the first three days of hospitalization. Laboratory parameters showed no evidence of renal recovery, with serum creatinine rising from 5.34 mg/dL to 5.67 mg/dL and serum phosphate increasing from 6.4 mg/dL to 7.04 mg/dL. Therefore, intermittent hemodialysis was initiated on hospital days 3 and 4.

Following dialysis and supportive treatment, the patient’s urine output gradually improved: 1,600 mL/day on day 4, 2,500 mL/day on day 5, and 3,800 mL/day on day 6. Serum creatinine levels progressively decreased from a peak of 5.67 mg/dL to 1.30 mg/dL by day 7 of hospitalization. Phosphate levels were normalized, and the patient remained hemodynamically stable after the treatment.

She was discharged in good clinical condition with substantially improved renal function and was advised to continue close outpatient nephrology follow-up. At a nephrology follow-up visit two weeks after discharge, renal function had further improved, and no additional hemodialysis was required. Serum creatinine had returned to near-baseline (0.74 mg/dL), and serum phosphate had normalized (3.98 mg/dL).

**Figure 3:**
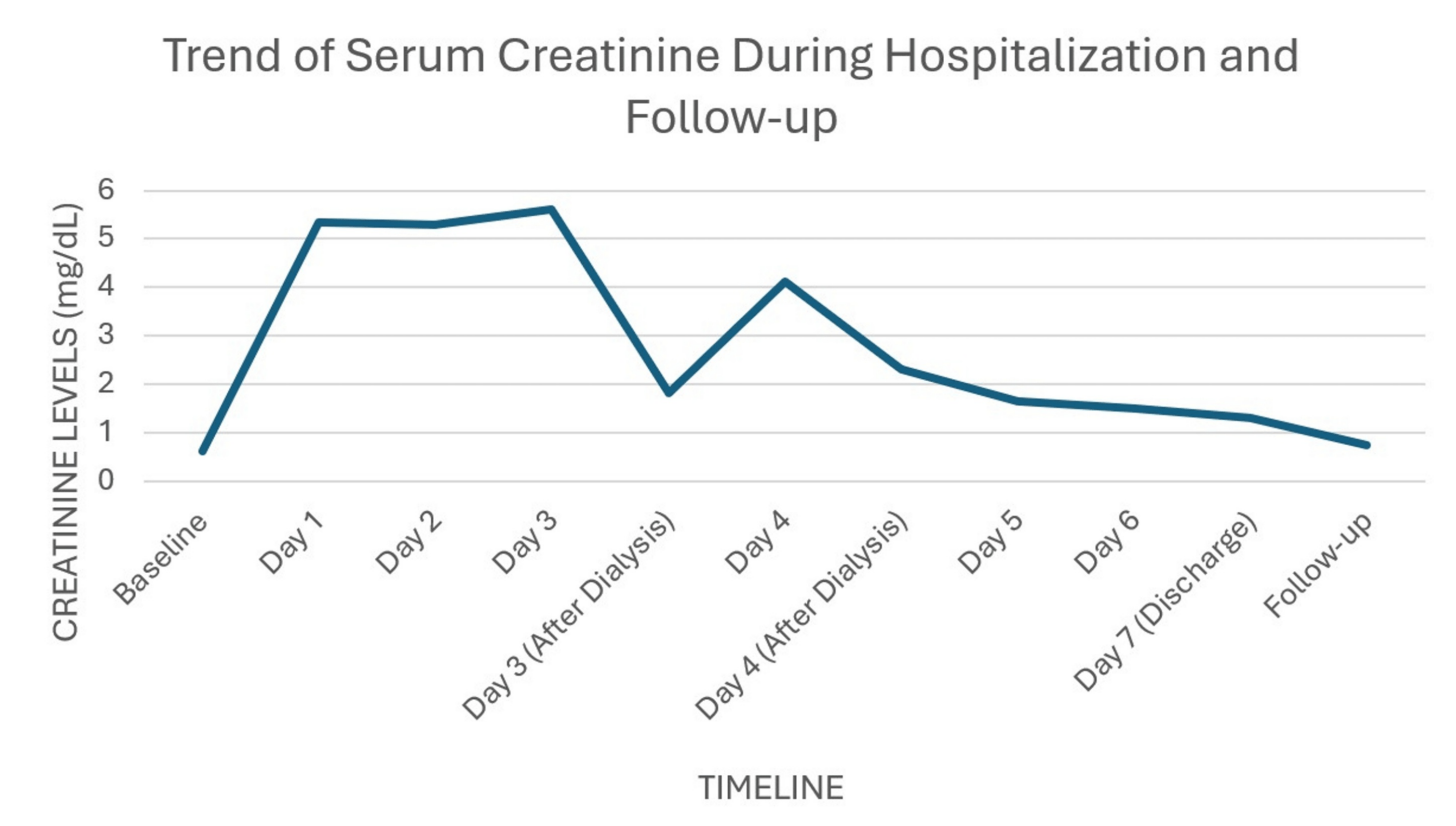
Trend of serum creatinine levels during hospitalization and follow-up, demonstrating progressive renal recovery following supportive treatment and temporary hemodialysis.

**Figure 4:**
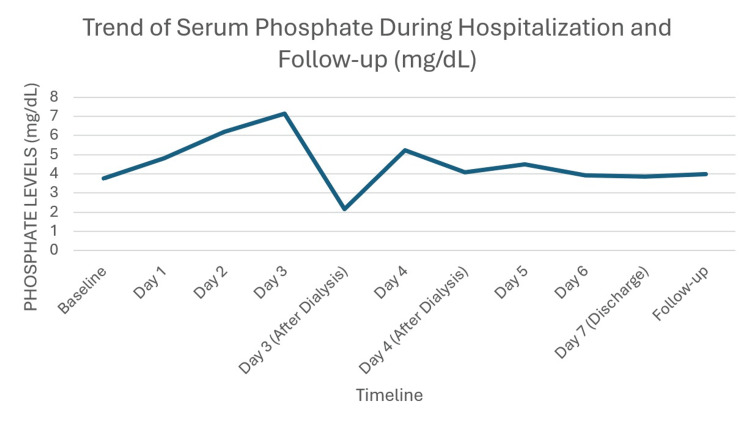
Trend of serum phosphate levels during hospitalization and follow-up, showing normalization after supportive treatment and temporary hemodialysis.

Figures 3-4 illustrate the serial changes in serum creatinine and phosphate levels during hospitalization and at the two-week follow-up after discharge.

## Discussion

APN is a rare but increasingly recognized cause of AKI. The pathophysiology involves excessive intestinal phosphate absorption prior to colonoscopy, leading to hyperphosphatemia and the deposition of calcium phosphate crystals in renal tubules, which eventually causes renal tubular injury [1]. Although the true incidence of APN remains uncertain because prospective studies are lacking, observational studies have consistently demonstrated an association between OSP bowel preparation and AKI. A large cohort study identified OSP exposure as the strongest independent risk factor for AKI (RR: 2.35, 95% CI: 1.51-3.66) [5], while subsequent studies showed that the risk is greatest in patients receiving ACE inhibitors or angiotensin receptor blockers and those with diabetes mellitus, with a significant decline in renal function following OSP exposure [6,7]. Collectively, these findings suggest that OSP-associated AKI occurs predominantly in patients with pre-existing risk factors, highlighting the importance of careful patient selection before OSP administration [5-7].

APN usually occurs insidiously; many patients develop AKI weeks or months after OSP exposure, frequently resulting in underdiagnosis [1,3,8]. Subclinical or delayed presentations remain a significant challenge, often obscuring the causal link to prior colonoscopy preparations [5-7]. Unlike this typical delayed progression, our patient presented with a rapid and severe clinical syndrome suggestive of APN, marked by sudden oliguria, grade 3 AKI, and hyperphosphatemia requiring urgent hospitalization.

In this patient, the concurrent administration of ramipril (an ACE inhibitor) and intermittent NSAID exposure (naproxen and dexketoprofen) may have contributed to renal hypoperfusion and increased susceptibility to AKI. The NSAID disrupts renal prostaglandin formation, impairing afferent arteriolar vasodilation, while the ACE inhibitor prevents efferent arteriolar vasoconstriction. This dual blockade disrupts renal autoregulation, reduces the glomerular filtration rate, and exacerbates tubular fluid stasis, creating a favorable microenvironment for rapid calcium-phosphate crystal deposition and subsequent acute tubular injury [1,2].

Renal biopsy remains the gold standard for confirming APN, but invasive procedures can be limited in acute clinical settings, depending on the patient's condition [1,2]. In this case, extensive non-invasive urine sediment analysis provided valuable supportive diagnostic information. The identification of pale, needle-shaped crystals under non-polarized light, combined with their characteristic birefringence under polarized light microscopy, provided morphological evidence of phosphate crystallization. This microscopic finding, together with the recent history of OSP exposure and the associated biochemical abnormalities, strengthened the clinical suspicion of APN. However, in the absence of renal biopsy, the diagnosis remained presumptive rather than histologically confirmed. Quantitative urinary phosphate measurements and urine pH were unavailable, representing an additional limitation of this report.

Phosphate-containing bowel preparations should be avoided in patients with predisposing factors, including diabetes mellitus, hypertension, dehydration, chronic kidney disease, or concurrent use of nephrotoxic medications [1,4]. Management of APN is primarily supportive and includes discontinuation of nephrotoxic agents, aggressive intravenous hydration, correction of electrolyte abnormalities, and renal replacement therapy when clinically indicated. In our patient, two hemodialysis sessions, along with supportive treatment, led to progressive renal recovery and normalization of serum phosphate levels. Similar outcomes have been described in previous reports, although some patients may progress to chronic kidney disease or end-stage kidney disease, particularly after repeated OSP exposure or delayed recognition [1,3,5,7]. Early recognition of suspected APN is important because prompt withdrawal of precipitating factors and supportive treatment may improve the likelihood of renal recovery.

## Conclusions

APN is a potentially serious and often underrecognized cause of AKI following phosphate-containing bowel preparation. Patients with risk factors such as chronic kidney disease, diabetes mellitus, hypertension, dehydration, and concurrent nephrotoxic medication use require careful evaluation before administration of OSP preparations. Early recognition of APN and careful evaluation of predisposing risk factors are essential to prevent irreversible renal injury.
